# A humanized trivalent Nectin-4-targeting nanobody drug conjugate displays potent antitumor activity in gastric cancer

**DOI:** 10.1186/s12951-024-02521-5

**Published:** 2024-05-16

**Authors:** Yue Wu, Min Zhu, Baihe Sun, Yongting Chen, Yuping Huang, Junwei Gai, Guanghui Li, Yanfei Li, Yakun Wan, Linlin Ma

**Affiliations:** 1 Shanghai University of Medicine & Health Sciences Affiliated Zhoupu Hospital, Shanghai, China; 2 School of Health Science and Engineering, University of Shanghai for Science and Technology, Shanghai, China; 3 Shanghai Novamab Biopharmaceuticals Co., Ltd., Shanghai, China; 4 Graduate School of Xinxiang Medical University, Henan, China

**Keywords:** Nectin-4, Nanobody drug conjugates, Gastric cancer

## Abstract

**Background:**

Gastric cancer represents a highly lethal malignancy with an elevated mortality rate among cancer patients, coupled with a suboptimal postoperative survival prognosis. Nectin-4, an overexpressed oncological target for various cancers, has been exploited to create antibody-drug conjugates (ADCs) to treat solid tumors. However, there is limited research on Nectin-4 ADCs specifically for gastric cancer, and conventional immunoglobulin G (IgG)-based ADCs frequently encounter binding site barriers. Based on the excellent tumor penetration capabilities inherent in nanobodies (Nbs), we developed Nectin-4-targeting Nb drug conjugates (NDCs) for the treatment of gastric cancer.

**Results:**

An immunized phage display library was established and employed for the selection of Nectin-4-specific Nbs using phage display technology. Subsequently, these Nbs were engineered into homodimers to enhance Nb affinity. To prolong in vivo half-life and reduce immunogenicity, we fused an Nb targeting human serum albumin (HSA), resulting in the development of trivalent humanized Nbs. Further, we site-specifically conjugated a monomethyl auristatin E (MMAE) at the C-terminus of the trivalent Nbs, creating Nectin-4 NDC (huNb26/Nb26-Nbh-MMAE) with a drug-to-antibody ratio (DAR) of 1. Nectin-4 NDC demonstrated excellent in vitro cell-binding activities and cytotoxic efficacy against cells with high Nectin-4 expression. Subsequent administration of Nectin-4 NDC to mice bearing NCI-N87 human gastric cancer xenografts demonstrated rapid tissue penetration and high tumor uptake through in vivo imaging. Moreover, Nectin-4 NDC exhibited noteworthy dose-dependent anti-tumor efficacy in in vivo studies.

**Conclusion:**

We have engineered a Nectin-4 NDC with elevated affinity and effective tumor uptake, further establishing its potential as a therapeutic agent for gastric cancer.

**Graphical abstract:**

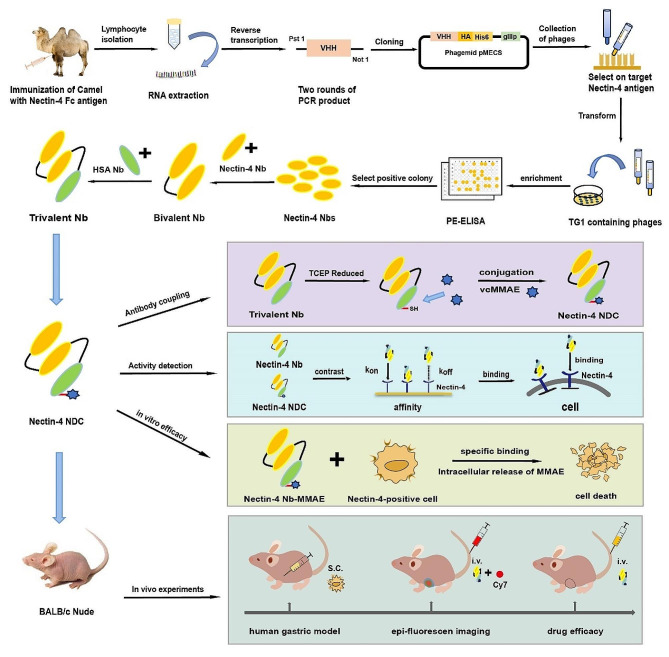

**Supplementary Information:**

The online version contains supplementary material available at 10.1186/s12951-024-02521-5.

## Introduction

Gastric cancer is recognized as the fifth most prevalent malignant tumor and the third leading cause of death among cancer patients [1]. The mortality rate associated with gastric cancer has markedly decreased in recent years, attributable to improved living conditions, early detection, and preventive measures [2]. Characterized by high molecular and phenotypic heterogeneity, gastric cancer is a complex disease and patients with advanced stages experience rapid deterioration and low surgical cure rates [2, 3]. In contemporary clinical practice, targeted medications play a pivotal role in reducing drug toxicity and enhancing patient survival rates [3, 4]. For instance, HER2-positive gastric cancer patients typically undergo first-line therapy with trastuzumab and platinum/5-Fluorouracil (5-FU) double agents [4]. Consequently, the development of targeted drugs for gastric cancer has emerged as a crucial strategy for addressing advanced cases.

Nectin-4/PVRL4 is a transmembrane cell adhesion molecule that activates the PI3K/AKT pathway, promoting tumor angiogenesis and participating in the growth, dissemination, and migration of tumor cells [5]. Initially identified as a receptor for the measles virus [5], Nectin-4 mediates viral entry into breast cancer and colorectal cancer cells through endocytic pathways, serving as a primary mediator of viral infection and spread [6]. Overexpressed in various cancers such as gastric, breast, lung, urothelial, and colorectal cancers, Nectin-4 is a validated clinical oncology target [7, 8]. Leveraging the biological characteristics of Nectin-4, antibody-drug conjugates (ADCs) developed targeting Nectin-4 have demonstrated significant efficacy in cancer treatment. Enfortumab Vedotin (Padcev), targeting Nectin-4, exhibited substantial therapeutic benefits in the EV-301 clinical trial for advanced urothelial cancer. Compared to the chemotherapy group, it reduced the risk of death by 30%, significantly extending overall survival, progression-free survival, and overall response rate [9]. It became the first FDA-approved targeted Nectin-4 ADC. Additionally, based on promising preclinical data, 9MW2821 and BT8009 are undergoing Phase I/II clinical trials in patients with advanced solid tumors [10, 11].

The evolution of ADCs has greatly expanded personalized tumor therapy, leveraging their unique structure for precise targeting and minimal off-target toxicity in ideal conditions. In the structural design of ADCs, the majority are either based on IgG design or created utilizing multivalent proteins [12], peptides [13, 14], multi-structured fusion proteins [15], and other small-molecule proteins [16] or mini-peptides [11]. However, in actual clinical research, the bind site barrier is a pivotal impediment hindering the development of ADCs [17]. Antibodies infiltrate the tumor mesenchyme, accumulating at the proximal binding site until saturation, leaving the distal site vacant [18]. This non-uniform distribution diminishes ADC therapeutic efficacy and may exacerbate tumor drug resistance [15, 18]. Overcoming the binding site barrier is crucial in ADC drug design to enhance efficacy. For instance, multiscale modeling accurately predicts ADC systemic distribution, informing tailored response strategies [19]. Another effective measure to address the binding site barrier is to adjust the size of antibody molecules in the ADC components [15]. Relevant studies suggest that proteins with smaller molecular weights can penetrate tissues more deeply, facilitating homogeneous binding to targets [20], and resulting in higher diffusion rates and uniform tissue distribution [21].

Nanobodies (Nbs), being the smallest naturally occurring antigen-binding fragments, possess stable structures and biological properties, enabling rapid production and facile engineering modifications [22]. Their highly homologous variable domains of the heavy chain to human antibodies make them readily suitable for humanization, with minimal immunogenicity concerns [23]. Moreover, their size and affinity kinetics can impact the targeting and penetration of solid tumors [20]. In 2018, the FDA approved Caplacizumab, the inaugural Nanobody worldwide, meticulously engineered for the precise targeting of vascular hemophilic factors in the management of acquired thrombotic thrombocytopenic purpura [24]. Subsequently, in 2022, the FDA authorized Ciltacabtagene autoleucel, a Nb-based chimeric antigen receptor T-cell (CAR-T) therapy designed to target B-cell maturation antigens for the treatment of relapsed or refractory multiple myeloma [25]. This innovative therapy exhibited profound and sustained efficacy throughout Phase Ib/II clinical investigations, maintaining its therapeutic benefits during a 2-year follow-up period [26, 27]. Apart from CAR-T, Nb can be utilized for delivering immunotoxins [28], and neutralizing viruses and toxins [29, 30], and has been studied in the fields of disease therapy, molecular imaging, and detection [31], demonstrating Nb’s significant potential. Nbs have been validated in numerous preclinical studies for developing anticancer Nb drug conjugates (NDCs) [16, 21, 32]. NDCs exhibit not only promising anti-tumor effects but also faster in vivo diffusion and tumor uptake compared to ADCs [11, 16, 21]. However, data regarding NDCs targeting Nectin-4 for anticancer purposes are currently unavailable. Furthermore, a primary consideration in developing Nb-based therapeutics is their relatively short blood half-life, given their molecular weight falls below the renal threshold for glomerular filtration (60 kDa). Currently, strategies such as Nb conjugation with Fc domains or binding to human serum albumin (HSA) are widely applied to extend their half-life [21, 33, 34].

In this study, we devised an innovative humanized trivalent Nb-based NDC targeting Nectin-4 and assessed its therapeutic efficacy in a human gastric cancer model. The molecular weight of trivalent Nb is a mere 42 kDa, comprising two homologous Nectin-4 Nbs and one high-affinity Nb (15 kDa) targeting HSA. Trivalent Nb is interconnected via maleimide linkers with valine-citrulline cleavable sites and coupled with a microtubule inhibitor, monomethyl auristatin E (MMAE), culminating in a drug-to-antibody ratio (DAR) of 1. We constructed Nectin-4 NDC (huNb26/Nb26-Nbh-MMAE) with a molecular weight of 43 kDa. Furthermore, employing imaging systems, we scrutinized the swift diffusion and heightened tumor uptake of Nectin-4 NDC in vivo. In the context of i*n vivo* efficacy assessments, Nectin-4 NDC demonstrated remarkable tumor inhibitory effects, suggesting its potential as a treatment for gastric cancer.

## Materials and methods

### Cell lines

From ATCC, BT474, NCI-N87, and HEK-293T were acquired. 10% FBS and 1% BT474 advance were added to DMEM (BI) for the growth of BT474 cells (cobioer, Nanjing, China). NCI-N87 cell was developed using RPMI 1640 media (BI) enhanced with 10% FBS (BI). The cell culture media were supplemented with 1% GlutaMAXTM-I (10,000 U/mL) (Gibco) and 1% penicillin-streptomycin (10,000 U/mL) (Gibco). The 293T-human-Nectin-4 is a stable cell line expressing human Nectin-4, achieved by amplifying the human Nectin-4 gene sequence, inserting it into the pLVX-EF1α-puro vector, and subsequently transfecting with lentivirus.

### Immunized phage display library establishment and Nectin-4 nb screening

To generate Nectin-4-Fc fusion proteins utilizing the HEK 293 F expression system, the Nectin-4 gene underwent extensive PCR amplification, was then inserted into the pFUSE vector (Invitrogen), and subsequently purified using protein A affinity chromatography. A male Bactrian camel received weekly immunizations with a blend of Nectin-4-Fc fusion protein and Fever’s adjuvant (Sigma-Aldrich). Following seven immunizations, 100 mL of camel blood was utilized for peripheral blood mononuclear cells (PBMCs) isolation, total RNA extraction, cDNA synthesis, and two rounds of PCR to generate variable domain of the heavy chain of heavy-chain antibody (VHH) fragments. For the establishment of a phage display library harboring the VHH gene, the VHH fragment inserted into the phagemid pMECS vector was transformed into E. coli. The library underwent screening using phage display technology. The 96 clones obtained underwent three rounds of screening and were enriched for analysis via periplasmic extract ELISA (PE-ELISA). Ultimately, sequence detection was employed to differentiate among families for the produced Nbs specifically targeting Nectin-4. Nectin-4 Nbs were predominantly produced in E. coli, followed by purification using protein A affinity chromatography (Qiagen), and the Nbs’ purity was assessed through SDS-PAGE. Throughout the entire process, the guidelines outlined in the national institutes of health’s “Guide for the Care and Use of Laboratory Animals” were strictly adhered to.

### Nectin-4 specific nbs selection through flow cytometry

NCI-N87 and BT474 cells were seeded in 96-well plates at a density of 2 × 10^5^ cells per well, followed by two washes with PBS. The purified Nectin-4 Nbs were diluted in PBS containing 2% FBS. Subsequently, 100 µL of the Nectin-4 Nbs solution was added to the 96-well plate, and the cells were incubated for 30 min at 4 °C. After two PBS washes, 100 µL of APC anti-HA.11 Epitope Tag was applied and incubated for an additional 30 min at 4 °C. Following two PBS washes, the cells were resuspended in 200 µL PBS and thoroughly mixed for flow cytometry analysis.

### Production and purification of multivalent Nectin-4 Nb

The bivalent Nectin-4 Nb consists of two homologous Nectin-4 Nbs, while the trivalent Nectin-4 Nb is composed of the bivalent Nectin-4 Nb and one HSA Nb. Maintain the CDR regions of the recombinant antibody sequence unchanged while humanizing its FR regions [35, 36]. Next, amplify the sequence and ligate it into the pPICZɑA vector. Subsequently, this vector was transformed into *Pichia pastoris* X-33 receptor cells to induce the expression of the multivalent Nectin-4 Nb using methanol. Following purification through protein A affinity chromatography, the purity of the multivalent Nectin-4 Nb was assessed using SDS-PAGE.

### Generation of Nectin-4 Nb drug conjugates

Trivalent Nectin-4 Nb was incubated in PBS buffer containing 10 mM TECP (Sigma-Aldrich) overnight at 4 °C and subsequently purified to eliminate excess TCEP. The TCEP-reduced Nbs were then subjected to a sequential reaction at room temperature with a double molar ratio of MC-VC-PABC-MMAE (vcMMAE) for 2 h. The process was terminated by the addition of 20-fold molar equivalents of acetylcysteine. Following purification, the NDC underwent buffer replacement with PBS. Disulfide bonds between antibody chains were oxidized using CuSO_4_ as an oxidizing agent [37]. Trivalent Nectin-4 Nb, both before and after TCEP reduction, as well as Nectin-4 NDC, were incubated for 2 h at room temperature with a 2 mM CuSO_4_ oxidant. The antibody coupling efficiency was assessed using SDS-PAGE under non-reducing conditions.

### Analysis of DAR

The average DAR of trivalent Nectin-4 Nb conjugated with vcMMAE was determined using ultraviolet-visible (UV-VIS) spectroscopy [38, 39]. The Nano300 full-wavelength ultramicro spectrophotometer (Allsheng) was employed to detect and record the absorbance values of huNb26/Nb26-Nbh, vcMMAE, and Nectin-4 NDC at 254 nm and 280 nm. These values were utilized to calculate the DAR of the NDC, with 254 nm representing the maximum absorption wavelength of vcMMAE. The extinction coefficients for calculating the DAR are documented in Supplementary Table (Additional file 1: Table S1).

### Antigen-binding activity at cell surfaces

NCI-N87 cells and 293T-human-Nectin-4 cells were plated at a density of 1 × 10^5^ cells per well in a 96-well plate and subsequently washed. huNb26/Nb26-Nbh and Nectin-4 NDC were diluted in PBS containing 2% FBS and added to the 96-well plate, followed by incubation with cells at 4℃ for 30 min. Following cell washing, 100 µL of goat poly-antibody (Novamab) was added to each well and incubated at 4℃ for 30 min. Subsequently, 100 µL of donkey anti-goat IgG H&L (Abcam) was added to each well, and the cells were incubated at 4℃ for another 30 min. Finally, the cells were resuspended for flow cytometry analysis.

### Bio-layer Interferometry (BLI)

The Fortebio Octet system (Fortebio) was utilized to assess the affinity of huNb26/Nb26-Nbh and Nectin-4 NDC for the recombinant human Nectin-4 antigen. Streptavidin A biosensors were equilibrated with PBST buffer for 10 min. Subsequently, the biosensor streptavidin A was exposed to diluted biotinylated Nectin-4 protein, and after mixing, it was immersed in PBST buffer. The biosensor was then subjected to association with a range of diluted huNb26/Nb26-Nbh or Nectin-4 NDC samples. The association rates (K_on_), dissociation rates (K_off_), and dissociation constants (K_d_) were analyzed utilizing ForteBio Data Analysis 9.0 software.

### *In vitro* drugs activity evaluation

Cells in the exponential growth phase were harvested and seeded at a density of 4 × 10^3^ cells per well into 96-well plates, followed by overnight incubation. The 96-well plate was filled with gradient dilutions of the NDC along with complete growth medium. Cells were incubated at 37 °C for 96 h. 20 µL of cell counting Kit-8 (Dojindo) was administered to the cells, which were then incubated at 37 °C for 4 h. After shaking the 96-well plates, absorbance was measured at 450 nm using an enzyme marker. Inhibition % = (1 – absorbance of experimental group) / average absorbance of blank group × 100. IC_50_ values were ascertained by non-linear regression analysis utilizing GraphPad Prism version 8.0.

### Anti-tumor effect of NDC in gastric mice xenograft models

Xenograft models derived from cell lines were employed to evaluate the in vivo anticancer efficacy of NDC. To establish a human gastric model, NCI-N87 cells were subcutaneously injected at the right flank of six-week-old female BALB/c nude mice, with a concentration of 5 × 10^6^ cells in 100 µL PBS mixed with Matrigel Matrix (MCE) at a 1:1 ratio. Once the tumor volumes reached 100 mm^3^, the mice were randomly divided into five groups (*n* = 8 per group). The positive drug group received 10 mg/kg 5-FU (MCE), while the negative control group was administered PBS. The experimental groups were treated with Nectin-4 NDC at doses of 2.5 mg/kg, 5 mg/kg, and 10 mg/kg. All groups receive medication every other day (Q2D) for a total of ten administrations. Subcutaneous tumor diameters were measured using a digital caliper, and tumor volumes were calculated using the formula 1/2 × (length × width^2^). Individual mouse tumor sizes were recorded and measured every two days. Ultimately, all mice were euthanized, and tumor tissues were excised and weighed. The Animal Experimental Ethics Committee at Shanghai University of Medicine and Health Sciences approved the mice studies.

### *In vivo* epi-fluorescence imaging experiments

To evaluate the NDC distribution in animals. Imaging was performed before and immediately after intravenous injection of 150 µg of Cy7-Nectin-4 NDC in mice bearing NCI-N87 human gastric cancer xenografts (*n* = 3), subsequent imaging was performed at 0.5, 3, 6, 24, and 48 h later. Three mice were euthanized after a 24-hour injection period, and their main organs were removed for imaging. The IVIS Lumina system (Caliper Life Science) was used to acquire images of three mice simultaneously, and the Living image software (Caliper LS) was utilized to analyze the data. ROIs were drawn around the tumor and organ areas, and the values of average fluorescence radiance (p/s/cm^2^/sr)/(µW/cm^2^) from these ROIs were used to calculate the distribution of NDCs to tumors and organs.

### H&E staining

Tumor tissue was removed following therapy, and preserved for 24 h by immersion in 4% paraformaldehyde, followed by embedding in paraffin. Slices measuring 4–5 μm were obtained using a microtome. After dewaxing in xylene, anhydrous ethanol, and 75% ethanol, the sections were washed with distilled water. Subsequently, the sections were dehydrated and stained with hematoxylin and eosin (H&E) for nuclear and cytoplasmic visualization, respectively. The processed tumor sections were then air-dried, rinsed in distilled water, dehydrated in anhydrous ethanol, and finally, sealed. Images were captured and documented using a PRECICE 500 digital section scanning machine (UNIC, China).

### Immunohistochemistry

The tumor tissue slices obtained previously underwent dewaxing and hydration. An endogenous peroxidase blocker was applied dropwise to the tissue to deactivate endogenous peroxidase. The sections were further subjected to antigen retrieval using an EDTA solution, involving two cycles of high-temperature heating and room-temperature cooling. After sealing with 5% BSA, the sections were treated overnight at 4 °C with appropriately diluted primary antibodies targeting cleaved caspase-3 and cleaved caspase-9. After a 30-minute warming step at 37 °C, the sections were exposed to a multimeric anti-mouse IgG-HRP secondary antibody for an additional 30 min at 37 °C. Color development was achieved using a DAB color solution. The cell nuclei were counterstained with Mayer’s hematoxylin, followed by sealing, and images were scanned and captured using the PRECICE 500 digital section scanner.

### Statistical analysis

Statistical analysis was carried out using GraphPad Prism version 8.0. Curves were fitted by non-linear regression. Data are expressed as EC_50_/IC_50_ ± SD or mean ± SEM. Student’s t test was used to measure differences between two groups, comparisons among multiple groups were performed using one-way ANOVA. *P* values were presented using the following asterisk rating system: *P* < 0.05 *, *P* < 0.01 **, *P* < 0.001 ***, and *P* < 0.0001 ****.

## Results

### Production of Nectin-4-targeting nbs

**Fig. 1:**
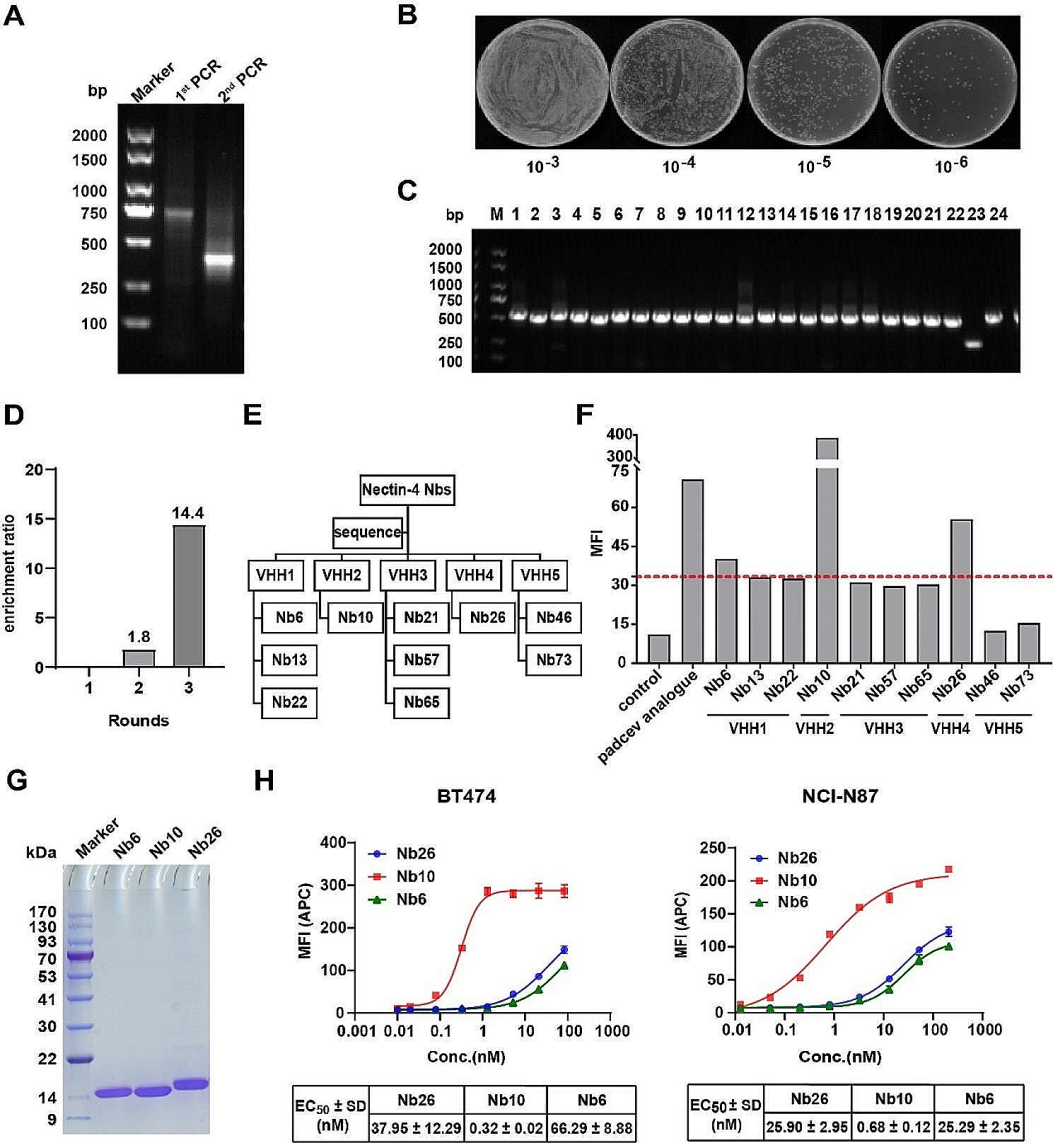
Production of Nectin-4-targeting monovalent Nbs. **A** VHH gene fragments were obtained through two rounds of PCR during the construction of the phage display library construction. **B** The size of the library was determined by counting the number of clones after serial dilutions and plating on plates containing selective antibiotics. **C** The insertion rate of the library was determined by performing PCR on randomly selected 24 colonies. **D** Folders containing enhanced clones in each cycle of phage display technology. **E** Classification of ten individually sequenced Nbs into distinct families. **F** Evaluation of the capacity of 10 differentially sequenced Nbs binding to the cell surface Nectin-4 by flow cytometry. **G** Detection of Nectin-4 Nb expression and purification using SDS-PAGE. **H** Flow cytometry detection of purified Nectin-4 Nbs binding activity to BT474 and NCI-N87 cells. Data are expressed as EC_50_ ± SD (*n* = 3)

To generate Nectin-4-targeting Nbs, Nectin-4-Fc antigen was produced using the HEK 293 F expression system and purified (Additional file 1: Figure S1A, B), and the Nectin-4-Fc protein displayed excellent binding activity, as demonstrated by ELISA (Additional file 1: Figure S1C). After immunizing camels with Nectin-4-Fc protein, blood was obtained, and through a series of procedures, a high-quality phage display library containing the VHH gene fragment was successfully constructed (Fig. 1A), boasting a library capacity of 6.15 × 10^9^ CFU (Fig. 1B), and a correct insertion rate of 95% (Fig. 1C). Conducting three rounds of phage display bio-panning, we efficiently screened for phages with specific binding activity, resulting in clones enriched up to 14.4 folds (Fig. 1D). From the enriched clones, 96 randomly selected clones underwent PE-ELISA, identifying 88 positive clones (Additional file 2: Figure S2). Following sequence detection, 10 Nbs from five families were chosen based on the complementarity determining region 3 (CDR3) of the VHH sequence (Fig. 1E). The screened Nbs demonstrated a specific capability to recognize and bind to the cell surface Nectin-4. Utilizing flow cytometry, we assessed the binding activity of these 10 Nbs with diverse sequences, revealing that the mean fluorescence intensity (MFI) values of Nb6, Nb10, and Nb26 were threefold higher compared to the control group values (Fig. 1F). As a result, we expressed and purified Nb6, Nb10, and Nb26 proteins, obtaining three Nbs of high purity through SDS-PAGE (Fig. 1G).

**Fig. 2:**
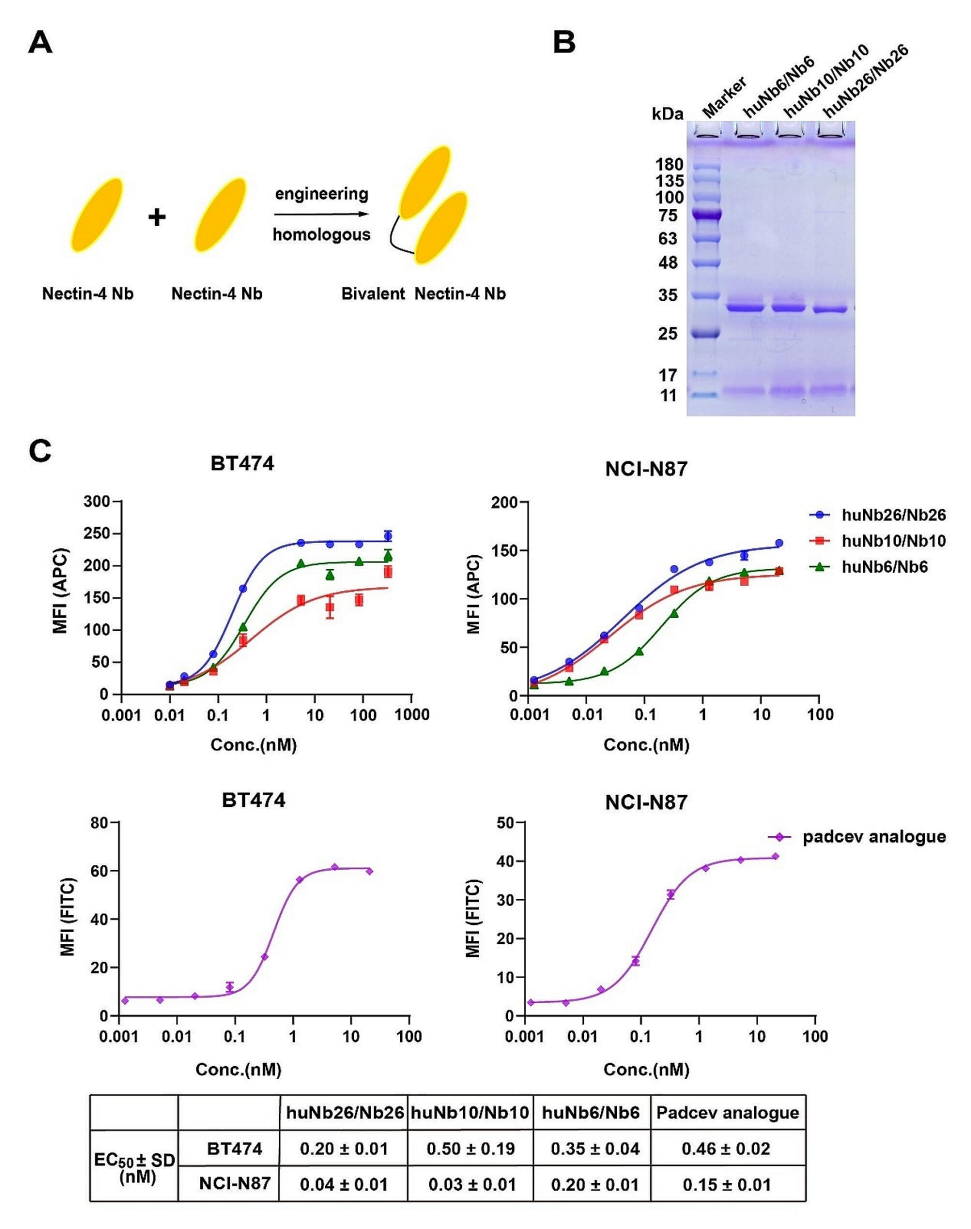
Investigation of binding activity of humanized homologous bivalent Nbs. **A** Schematic structure of bivalent Nbs. **B** Protein expression of bivalent Nbs was investigated utilizing SDS-PAGE. **C** The binding activity of bivalent Nbs on BT474 and NCI-N87 cells was assessed using flow cytometry. Data are expressed as EC_50_ ± SD (*n* = 3)

We further investigated the binding activity of three purified Nbs to BT474 and NCI-N87 cells, which inherently express Nectin-4, utilizing flow cytometry. The results indicated that monovalent Nectin-4 Nbs overall displayed low binding activity (Fig. 1H). To improve the binding ability of Nb, we developed three homologous bivalent Nbs that were humanized to minimize potential immunogenicity (Fig. 2A). Following production using the *Pichia* yeast expression system and purification, the binding activity to BT474 and NCI-N87 cells was significantly enhanced (Fig. 2B, C). Wherein, huNb26/Nb26, exhibited higher binding activity to both cells compared to the other two bivalent Nbs and demonstrated significantly better EC_50_ values than the Padcev analogue.

### Production of Nectin-4-targeted trivalent humanized NDC

**Fig. 3:**
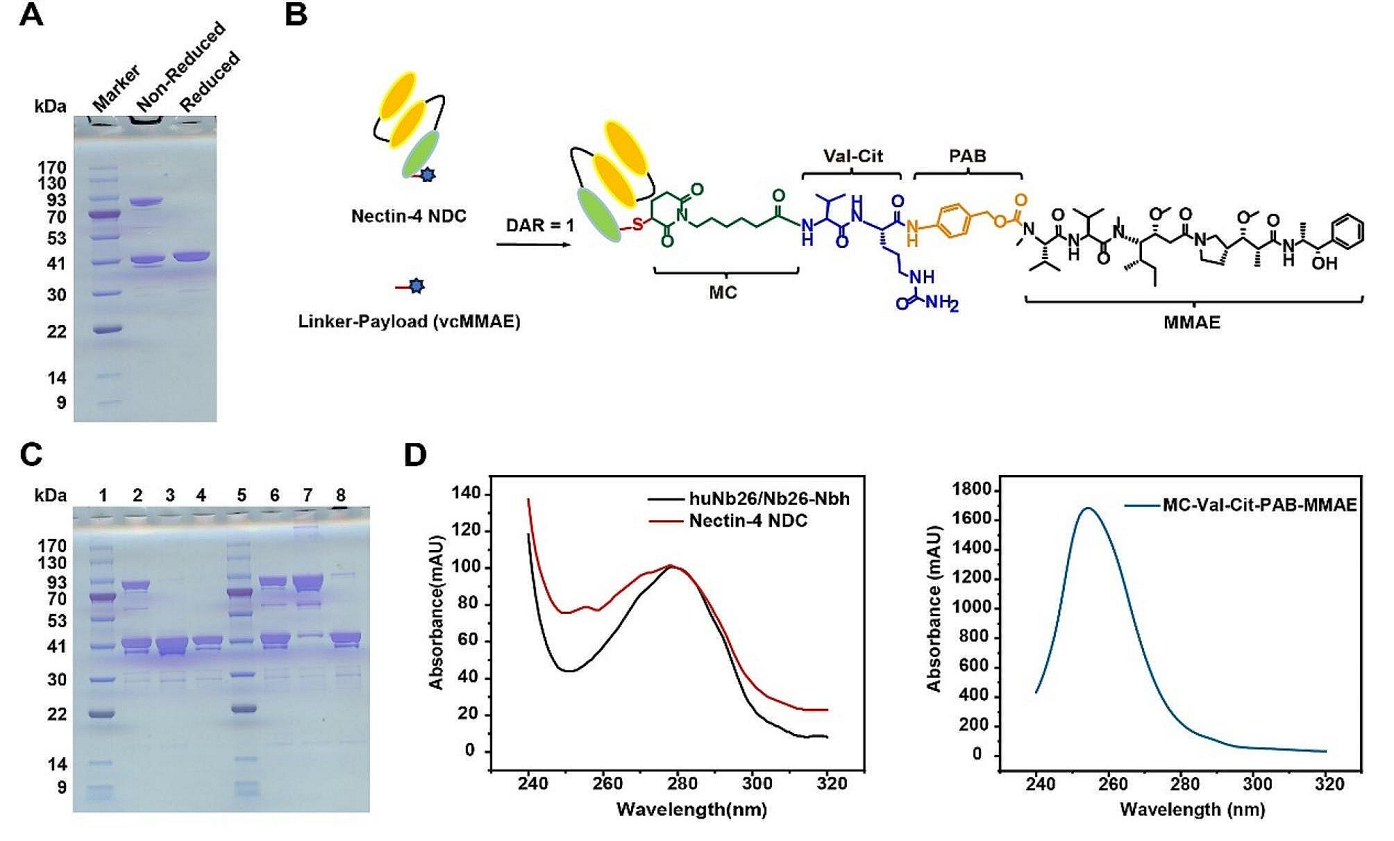
Production of Nectin-4-targeted trivalent humanized NDC. **A** Assessment of trivalent Nb purification and protein expression through SDS-PAGE. **B** Schematic representation of the Nectin-4 NDC structure. **C** SDS-PAGE analysis of Nectin-4 NDC under unreduced conditions. CuSO_4_ conditions without (2–4) and with (6–8) conditions, 1 and 5: marker, 2 and 6: huNb26/Nb26-Nbh, 3 and 7: TCEP-reduced huNb26/Nb26-Nbh, and 4 and 8: Nectin-4 NDC. **D** Absorption spectra of Nectin-4 NDC and huNb26/Nb26-Nbh, normalized at 280 nm (left), and the vcMMAE absorption spectra were used for conjugate (right)

We chose huNb26/Nb26, which exhibited the highest binding activity among the three bivalent Nbs. To prolong its in vivo half-life, we engineered huNb26/Nb26-Nbh by conjugating an HSA-targeted Nb to huNb26/Nb26. This design accounted for the rapid elimination of smaller Nb molecules through metabolic processes within the body. Additionally, a reactive cysteine was introduced at the C-terminus of huNb26/Nb26-Nbh for coupling drugs. Subsequently, huNb26/Nb26-Nbh was produced using the *Pichia* yeast expression system and purified (Fig. 3A). To ensure the druggability of the NDC, we selected the same valine-citrulline site and MMAE payload as those used in Padcev and 9MW2821. TCEP-reduction was employed to convert huNb26/Nb26-Nbh from a dimer to a monomer. Following this, the microtubule protein inhibitor MMAE was coupled to the C-terminal free cysteine site through a maleimide linker. Using this homogeneous coupling strategy, Nectin-4 NDC was produced, with a DAR of 1 on the structure (Fig. 3B).

After that, we employed the CuSO_4_ oxidation function to evaluate the conjugation of Nectin-4 NDC. SDS-PAGE analysis demonstrated that, in the absence of CuSO_4_, both Nectin-4 NDC and huNb26/Nb26-Nbh existed in the monomeric state. Conversely, in the presence of CuSO_4_, the majority of the NDC remained monomeric, while CuSO_4_ oxidized huNb26/Nb26-Nbh back to dimer under the same conditions (Fig. 3C). We identified the absorption peak of vcMMAE at 254 nm through UV-VIS spectroscopic analysis. Absorption spectra of Nectin-4 NDC and huNb26/Nb26-Nbh normalized at 280 nm, revealed higher absorbance at 254 nm for Nectin-4 NDC compared to huNb26/Nb26-Nbh (Fig. 3D). The produced Nectin-4 NDC exhibited an average DAR of 0.9. These data indicate the successful construction of Nectin-4 NDC with high coupling efficiency.

### *In vitro* activity and potency of Nectin-4 NDC

**Fig. 4:**
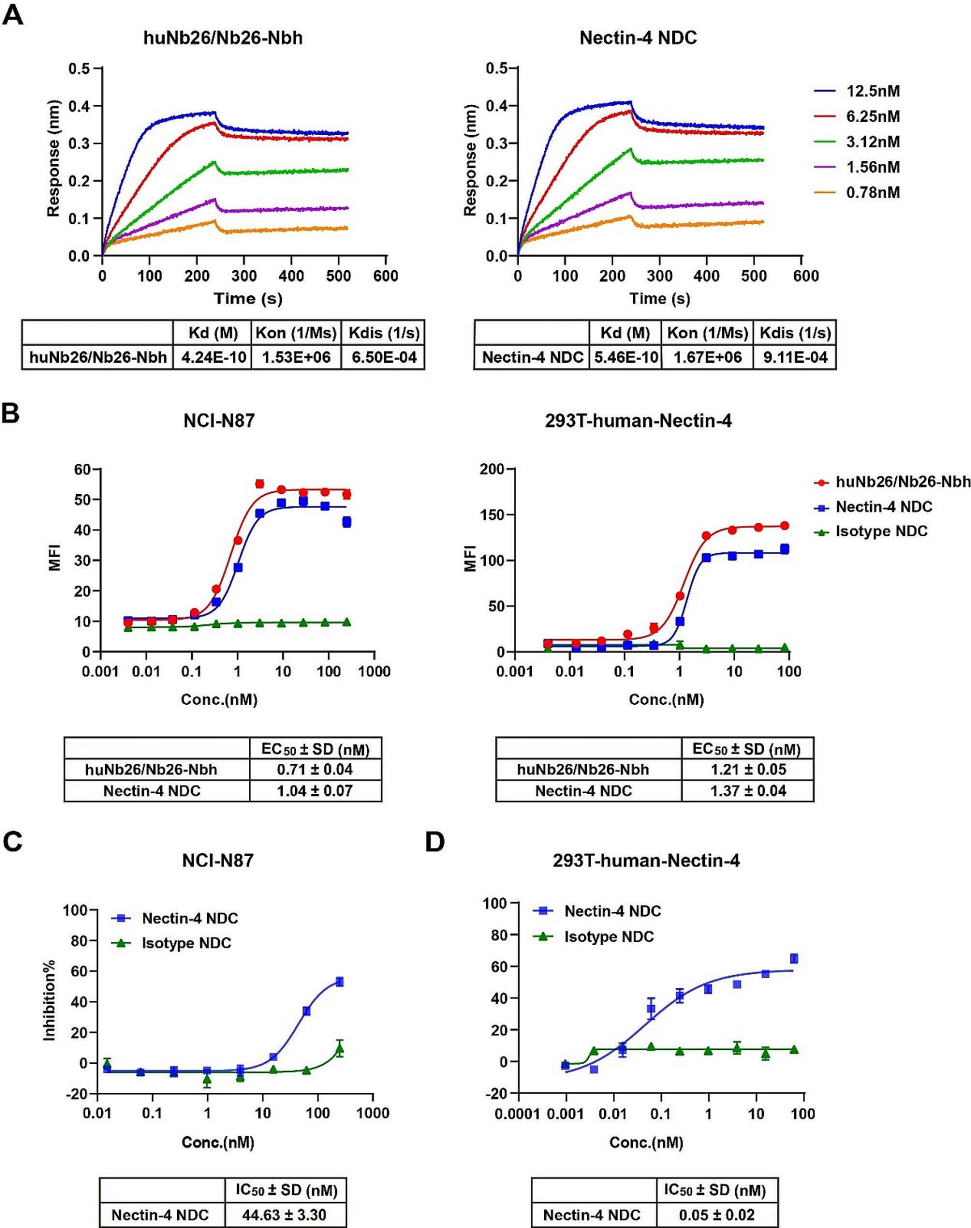
In vitro activity and potency of Nectin-4 NDC. The affinity of huNb26/Nb26-Nbh and Nectin-4 NDC was detected by BLI. **B** The binding activities of huNb26/Nb26-Nbh and Nectin-4 NDC were measured through flow cytometry. **C-D**In vitro inhibition rates of Nectin-4 NDC on NCI-N87 (**C**) and 293T-human-Nectin-4 (**D**) cells were evaluated employing the CCK-8 assay. Data are expressed as EC_50_/IC_50_ ± SD (*n* = 3)

To elucidate the affinity of Nbs and assess the potential impact of vcMMAE on Nb affinity, we evaluated the binding kinetics of huNb26/Nb26-Nbh and Nectin-4 NDC utilizing BLI. As depicted in Fig. 4A, both of them exhibited remarkably high affinities, with K_d_ values of 4.24 × 10^− 10^ M and 5.46 × 10^− 10^ M, respectively. Subsequently, we established an overexpression cell strain 293T-human-Nectin-4 (Additional file 3: Figure S3). Flow cytometry analysis of the cell-binding capabilities of Nectin-4 NDC and huNb26/Nb26-Nbh on 293T-human-Nectin-4 and NCI-N87 cells revealed robust cell-binding activities, with comparable EC_50_ values (Fig. 4B).

Subsequently, we assessed the tumor cell-killing effect using CCK8 assays, revealing that Nectin-4 NDC exhibited approximately 60% inhibition against NCI-N87 (Fig. 4C). Consistent with expectations, when the experiment was conducted on the overexpression cell line 293T-human-Nectin-4, the outcomes demonstrated 70% inhibition at nanomolar concentrations and an IC_50_ value of 0.05 nM (Fig. 4D). The enhanced inhibitory effect further underscores the positive pharmacological efficacy of Nectin-4 NDC.

### *In vivo* distribution and tumor uptake of Nectin-4 NDC

**Fig. 5:**
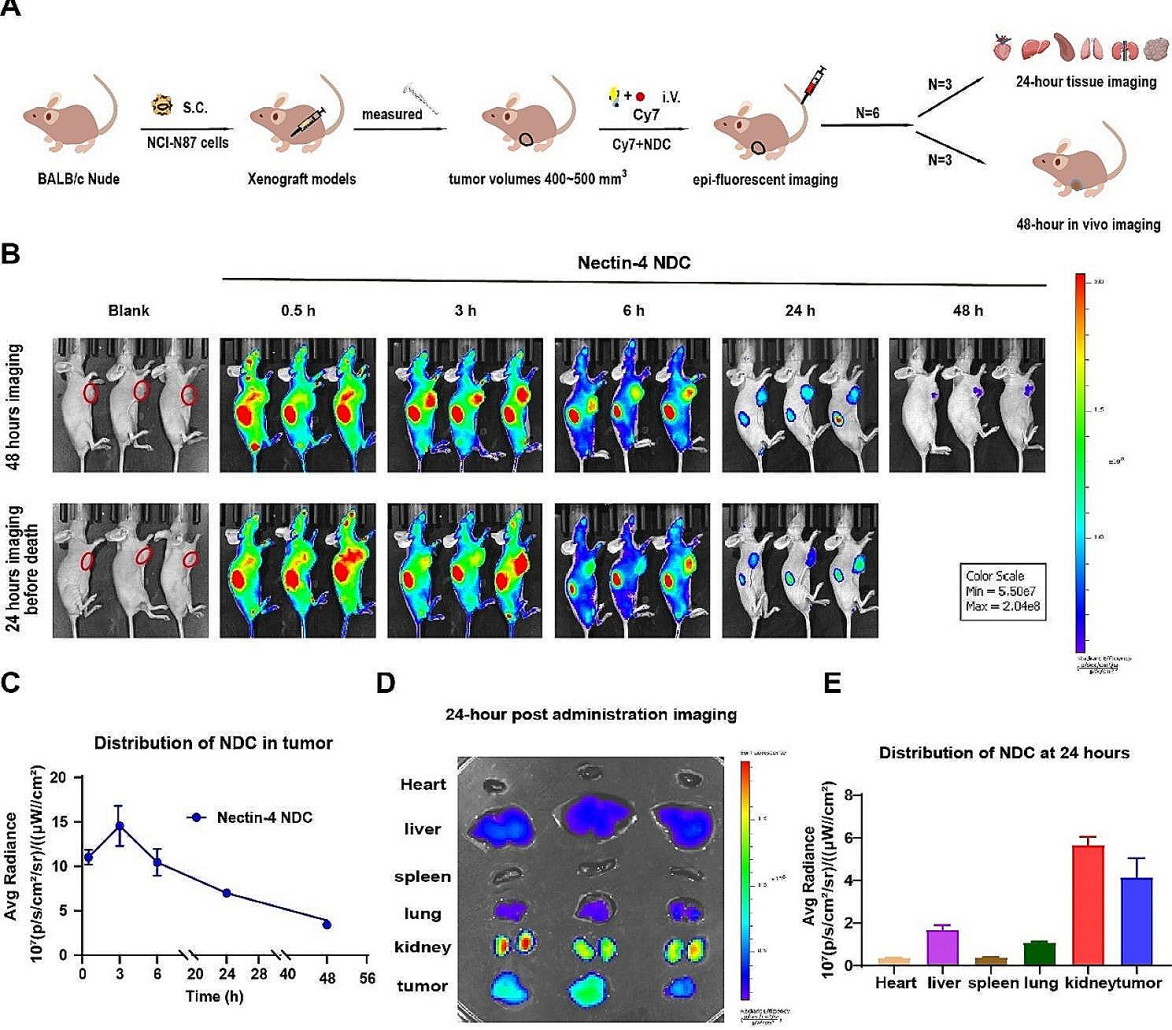
In vivo distribution and tumor uptake of Nectin-4 NDC. **A** Epi-fluorescent imaging was employed to assess the tumor uptake and distribution of Nectin-4 NDC in 6 BALB/c nude mice bearing NCI-N87 human gastric cancer xenografts. **B** Following intravenous injection of Cy7-labelled Nectin-4 NDC, the mice were scanned using the IVIS Lumina system at different time points after anesthesia. **C** The average fluorescence radiance values (p/s/cm^2^/sr)/(µW/cm^2^) for B were calculated using the Living image program (*n* = 3). **D** Fluorescence distribution in major organs at 24 h post-injection. **E** Quantitative analysis of the average fluorescence intensity in D using Living Image software (*n* = 3)

To acquire a more profound understanding of the in vivo targeting efficacy of Nectin-4 NDC, we conducted in vivo fluorescence imaging of Nectin-4 NDC in 6 BALB/c nude mice bearing NCI-N87 human gastric cancer xenografts (Fig. 5A). Following intravenous injection of Cy7-labelled Nectin-4 NDC into the mice, as illustrated in Fig. 5B, the fluorescence exhibited widespread distribution throughout the bodies of the mice at 0.5 h, with a high prominent concentration in the kidney region, which serves as the primary organ for Nb elimination. At 3 h post-injection, substantial fluorescence of Nectin-4 NDC accumulated at the tumor location was observed. From 6 to 24 h, the full body fluorescence of mice was steadily decreased, with residual fluorescence predominantly distributed on the tumor site and kidney. Even at 48 h, there was still minimal fluorescence detected at the tumor site. These findings indicate that Nectin-4 NDC exhibits high in vivo tumor uptake. Analyzing the average radiant efficiency in the tumor region revealed that Nectin-4 NDC achieved peak accumulation at the tumor site at 3 h, followed by gradual clearance and significant enrichment (Fig. 5C).

On the other hand, at 24 h after drug administration, three mice were anesthetized and executed for tissue imaging. Fluorescence predominantly accumulated in the tumor and kidney sites, with comparatively minimal presence in the liver and lungs, as depicted in Fig. 5D and E, consistent with the observations from the in vivo imaging.

### *In vivo* anti-tumor effect of Nectin-4 NDC

**Fig. 6:**
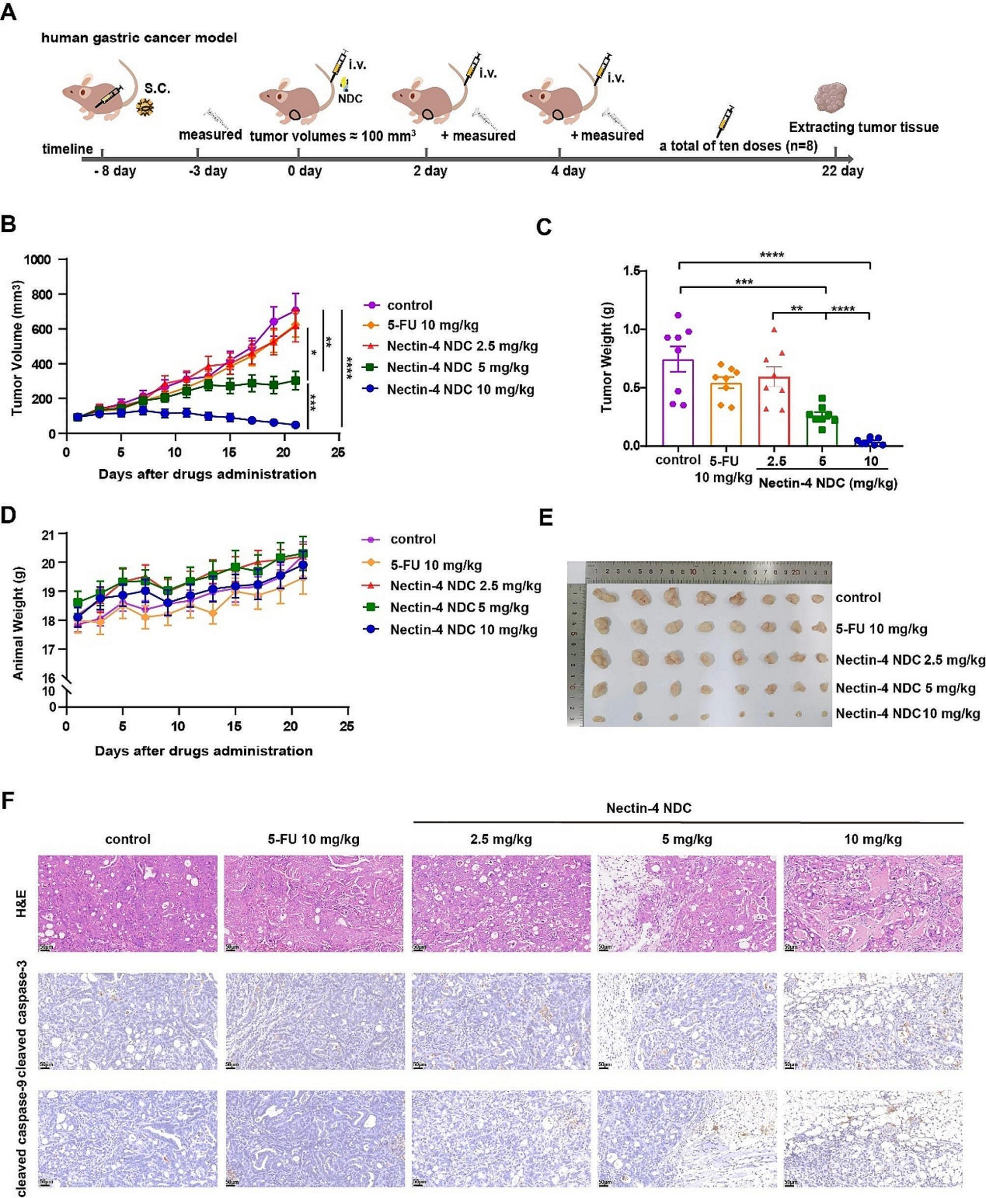
In vivo anti-tumor effect of Nectin-4 NDC. **A** Schematic representation of efficacy studies for Nectin-4 NDC in mice bearing NCI-N87 human gastric cancer xenografts. **B** Tumor volume changes during ten treatments (*n* = 8). **C** Weight of tumor (*n* = 8). **D** Body weight changes of mice during the treatment period (*n* = 8). **E** Tumor tissues were obtained through dissected mice in all groups after ten treatments. **F** H&E staining of tumor tissues and immunohistochemistry for cleaved caspase-3 and cleaved caspase-9 (*n* = 3), The representative image is shown. Data are expressed as mean ± SEM. *P* values were presented using the following asterisk rating system: *P* < 0.05 *, *P* < 0.01 **, *P* < 0.001 ***, and *P* < 0.0001 ****

Following the in vivo distribution study, we proceeded to evaluate the in vivo efficacy of Nectin-4 NDC, utilizing BALB/c nude mice bearing NCI-N87 human gastric cancer xenografts. When the average tumor volume approached 100 mm^3^, the treatment group received doses of 10 mg/kg, 5 mg/kg, and 2.5 mg/kg of Nectin-4 NDC (Fig. 6A). The positive-control group was given a dose of 10 mg/kg [40] of 5-FU, a traditional antitumor drug used to treat gastric cancer. During the subsequent 22-day treatment period, the drug was administered once every two days, with regular assessments of tumor volume measurements. As illustrated in Fig. 6B, C, and E, the treatment groups demonstrated a dose-dependent inhibition of tumor growth, with the high, medium, and low dose groups achieving mean tumor inhibition rates of 100%, 65.66%, and 14.06%, respectively. In the third delivery, the high-dose group (10 mg/kg) demonstrated substantial inhibition, maintaining the average tumor volume below 140 mm^3^. Subsequent treatments led to complete tumor regression. During the final week of treatment, the medium-dose group (5 mg/kg) significantly reduced tumor growth. The low-dose group (2.5 mg/kg) did not exhibit significant tumor inhibition compared to the control group but displayed a similar tumor decline as the 10 mg/kg 5-Fu treated group. Additionally, the mice did not exhibit any adverse reactions to the drugs, such as loss of weight (Fig. 6D), weakness, or pain. Throughout the study period, the mice exhibited strong antitumor efficacy and showed tolerance to the drug.

We further utilized H&E staining and immunohistochemistry to study the morphological changes induced by Nectin-4 NDC in gastric cancer. In contrast to the control group, tumor tissues in the Nectin-4 NDC-treated group exhibited morphological alterations, including significant tissue vacuolization, nucleolysis, decreased cell number, and loss of cellular structure as evidenced by H&E staining (Fig. 6F). We assessed the expression of cleaved caspase-3 and cleaved caspase-9, which exhibited increased levels in the Nectin-4 NDC-treated group (Fig. 6F). These observations suggest that Nectin-4 NDC induce apoptosis in gastric cancer cells.

## Discussion

Nectin-4, as a cancer biomarker, has been elucidated to be intricately associated with lymph node metastasis mediated through the PI3K/AKT pathway, ultimately leading to an unfavorable prognosis, with its expression closely correlated with different TNM stages of gastric cancer [8, 41]. Therefore, Nectin-4 holds promise as a hopeful therapeutic target for gastric cancer. The successful preclinical trials of Padcev for treating urothelial carcinoma have demonstrated the efficacy of targeting Nectin-4 in cancer drug development [42], concurrently propelling the advancement of Nectin-4 ADCs. Given the high production costs, limited tissue penetration, binding site barriers, and off-target toxicity associated with monoclonal antibodies (mAb)-based ADCs, small-sized carriers, such as antigen-binding peptides [11], scFv [38, 43], Fab [44, 45], and Nb [16, 21], have been developed to address the narrow therapeutic window of ADCs. Among these, Nb stands out for its advantages, including a small molecular size, high stability, low immunogenicity, and cost-effectiveness. Leveraging Nb, we developed a novel Nectin-4-targeting NDC and evaluated its anti-tumor efficacy against gastric cancer. Our in vivo experiments yielded promising results, suggesting that the Nectin-4 NDC could be an effective strategy for treating gastric cancer.

As of 2023, among the 15 ADCs approved for marketing, 6 incorporate MMAE or MMAF [46]. MMAE functions by inhibiting microtubule polymerization, thereby further suppressing cell mitosis, inducing cell cycle arrest, and ultimately leading to tumor cell apoptosis, achieving therapeutic efficacy in tumor treatment [47]. Consequently, Nectin-4 ADCs may exhibit therapeutic effects comparable to ADCs in clinical settings. Utilizing the widely adopted site-specific conjugation approach, Nectin-4 ADCs maintain a uniform DAR, addressing concerns about the narrow therapeutic window linked to off-target toxicity in ADC treatments [37]. Given that Nbs, in contrast to mAb, have fewer conjugation sites, we intentionally opted for a DAR identical to that of n501-SN38 [16] and BT8009 [11] during MMAE conjugation. Subsequent studies on affinity and binding activity revealed that Nectin-4 ADCs engineered through this strategy remain unaffected by MMAE, exhibiting extremely high affinity and cell binding activity at the nanomolar level.

The results of our in vivo efficacy study, conducted on nude mice carrying NCI-N87 human tumor xenografts, indicate that the high dose (10 mg/kg) of Nectin-4 NDC achieved complete tumor regression, displaying a dose-dependent trend. During the treatment, there were no instances of drug toxicity-related deaths or signs of drug intolerance such as weakness or weight loss observed in the mice. However, in our in vitro efficacy studies, Nectin-4 NDC did not exhibit 100% cytotoxicity on NCI-N87 gastric cancer cells expressing Nectin-4. This discrepancy may be attributed to two factors. Firstly, it could be related to the quantity of Nectin-4 expressed in tumor cells. Previous research with 9MW2821 demonstrated significant cytotoxicity in cell lines with high Nectin-4 expression, but had a less pronounced effect on cell lines with medium to low Nectin-4 expression [10]. Our subsequent research further supported this observation, showing an enhanced cytotoxic effect of Nectin-4 NDC in cells overexpressing Nectin-4. Secondly, this inconsistency may be associated with the absence of the tumor microenvironment in the in vitro setting. In vivo, following the interaction of ADCs with the target antigen expressed on cancer cells and the release of MMAE, besides directly inducing cancer cell death, it can further trigger immunogenic cell death (ICD), ultimately activating the immune response in the tumor microenvironment [48, 49]. The ADC SGN-35 (Brentuximab vedotin), conjugated with vcMMAE and targeting CD30, induced endoplasmic reticulum stress after releasing MMAE, leading to further ICD-dependent cytotoxicity and ultimately activating anti-tumor immune responses [50]. The notable efficacy observed in our in vivo study may be associated with this mechanism.

The outstanding tumor-targeting and rapid tumor uptake of Nbs have been extensively validated [16, 21, 51], and the conjugation of Nbs with near-infrared fluorescence holds potential as a specialized tracer for cancer imaging [52]. In our study, Cy7-labeled Nectin-4 NDC exhibits rapid systemic tissue distribution and a high level of tumor uptake in vivo, with an extended residence time at the tumor site compared to their retention in other organs. Conversely, administration of free Cy7 dye in mice results in rapid clearance, with the non-targeted Cy7-labeled control showing minimal tumor accumulation [53, 54]. Therefore, theoretically, the inclusion of Cy7 dye does not impact the specific tumor-targeting capability of Nectin-4 NDC.

Due to the non-recognition of murine albumin by our HSA Nb, the majority of Nectin-4 NDCs are cleared through renal elimination within 48 h. In our previous study, trivalent Nbs containing HSA Nb demonstrated a prolonged residence time of up to 12 days in cynomolgus monkeys [55]. Consequently, our Nectin-4 NDCs may potentially maintain a comparable half-life in cynomolgus monkeys and the human body. We opted for the incorporation of the HSA mechanism to extend the half-life of Nb, similar to the approach involving the Fc domain binding [56, 57]. Both mechanisms achieve a prolonged half-life through binding to FcRn, thereby avoiding lysosomal degradation. However, the binding of Nectin-4 NDC to HSA effectively avoids adverse reactions, such as thrombocytopenia associated with the binding of the Fc domain to Fcγ receptors [56, 57].

Furthermore, in our in vivo efficacy studies, we observed that during the initial week of treatment, the tumor inhibitory effect in the 5-FU treated positive control group resembled that of the medium-dose Nectin-4 NDC treated group (5 mg/kg). In the later stages, the inhibitory effect was comparable to the low-dose group (2.5 mg/kg), exhibiting no significant difference from the control group. This phenomenon may be linked to the resistance observed against 5-FU [58, 59]. The study suggests that our Nectin-4 NDC may exhibit efficacy in tumors resistant to 5-FU. Addressing the treatment of resistant tumors by Nectin-4 NDC will be a key focus in our subsequent research.

## Conclusions

In this study, we developed a novel Nectin-4-targeting Nb drug conjugate comprising two identical Nectin-4 Nbs, an HSA Nb, and vcMMAE, with homogeneous DAR, favorable affinity, and significant tumor uptake. Our Nectin-4 NDC effectively inhibited gastric cancer growth in vivo in a dose-dependent manner, indicating its potential as an effective anti-tumor agent for gastric cancer and other Nectin-4-positive tumors.

### Electronic supplementary material

Below is the link to the electronic supplementary material.

Supplementary Material 1

## Data Availability

No datasets were generated or analysed during the current study.

## Abbreviations

ADCs: Antibody drug conjugates
IgG: Immunoglobulin G
Nbs: Nanobodies
NDCs: Nanobody-drug conjugates
5-FU: 5-Fluorouracil
HSA: Human serum albumin
MMAE: Monomethyl auristatin E
DAR: Drug-to-antibody ratio
PE-ELISA: Periplasmic Extract ELISA
mAb: Monoclonal antibodies
vcMMAE: MC-VC-PABC-MMAE
UV-VIS: Ultraviolet-visible
Kon: Association rates
Koff: Dissociation rates
Kd: Dissociation constants
PBMCs: Peripheral blood mononuclear cells
VHH: Variable domain of the heavy chain of heavy-chain antibody
BLI: Bio-layer interferometry
H&E: Hematoxylin and eosin
CDR3: Complementarity determining region 3
MFI: Mean fluorescence intensity
ICD: Immunogenic cell death
