# Supplementary material for: A humanized trivalent Nectin-4-targeting nanobody drug conjugate displays potent antitumor activity in gastric cancer
Source: J Nanobiotechnology. 2024 May 16;22:256. doi: 10.1186/s12951-024-02521-5 (PMC11097425; doi:10.1186/s12951-024-02521-5)
Supplement: Supplementary file 1 — Supplementary Material 1 [file 12951_2024_2521_MOESM1_ESM.docx]

**Supplementary information**

Additional file 1: Table S1. Extinction coefficients are used for calculating DAR.

Additional file 2: Figure S1. Preparation of Nectin-4-Fc antigen. **A** The plasmid containing the Nectin-4 gene was transfected into 293F cells for expression, purified by affinity chromatography, and utilized for camel immunization. **B** Expression of the Nectin-4-Fc antigen was confirmed via SDS-PAGE. **C** The binding activity of the Nectin-4-Fc antigen was validated through ELISA.

Additional file 3: Figure S2. 88 positive clones were identified through PE-ELISA out of 96 randomly selected clones.

Additional file 4: Figure S3. 293T-human-Nectin-4, which stably expresses the Nectin-4 receptor, was constructed while its over-expression function was confirmed by flow cytometry.

**Table S1** Extinction coefficients are used for calculating DAR.

| Molecule | Extinction Coefficients (cm^−1^M^−1^) Calculated at Given Wavelength | |
| --- | --- | --- |
|  | 254 nm | 280 nm |
| huNb26/Nb26-Nbh | 21159 | 46087 |
| MC-Val-Cit-PAB-MMAE | 16840 | 2260 |


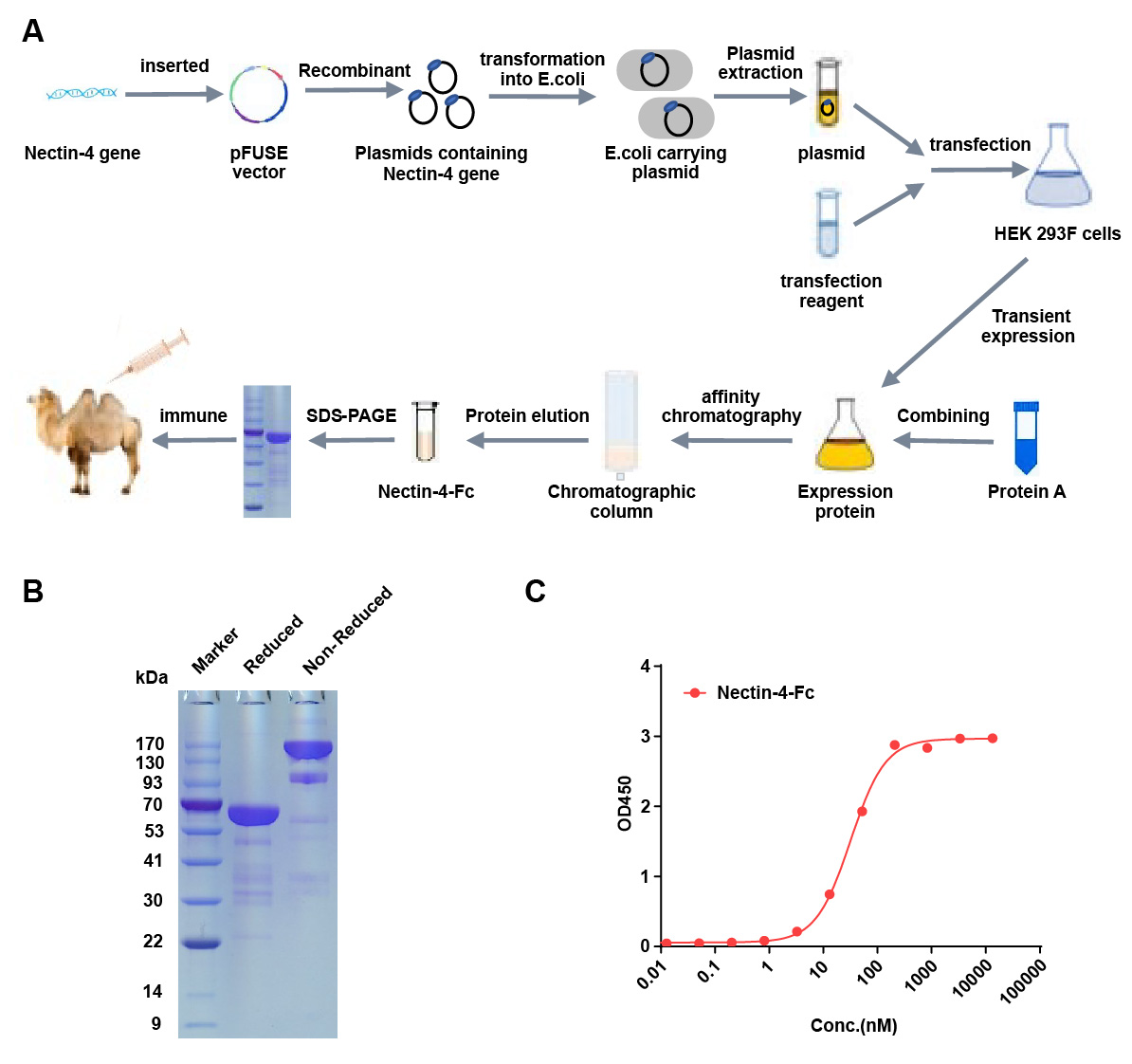


**Figure S1** Preparation of Nectin-4-Fc antigen. **A** The plasmid containing the Nectin-4 gene was transfected into 293F cells for expression, purified by affinity chromatography, and utilized for camel immunization. **B** Expression of the Nectin-4-Fc antigen was confirmed via SDS-PAGE. **C** The binding activity of the Nectin-4-Fc antigen was validated through ELISA.


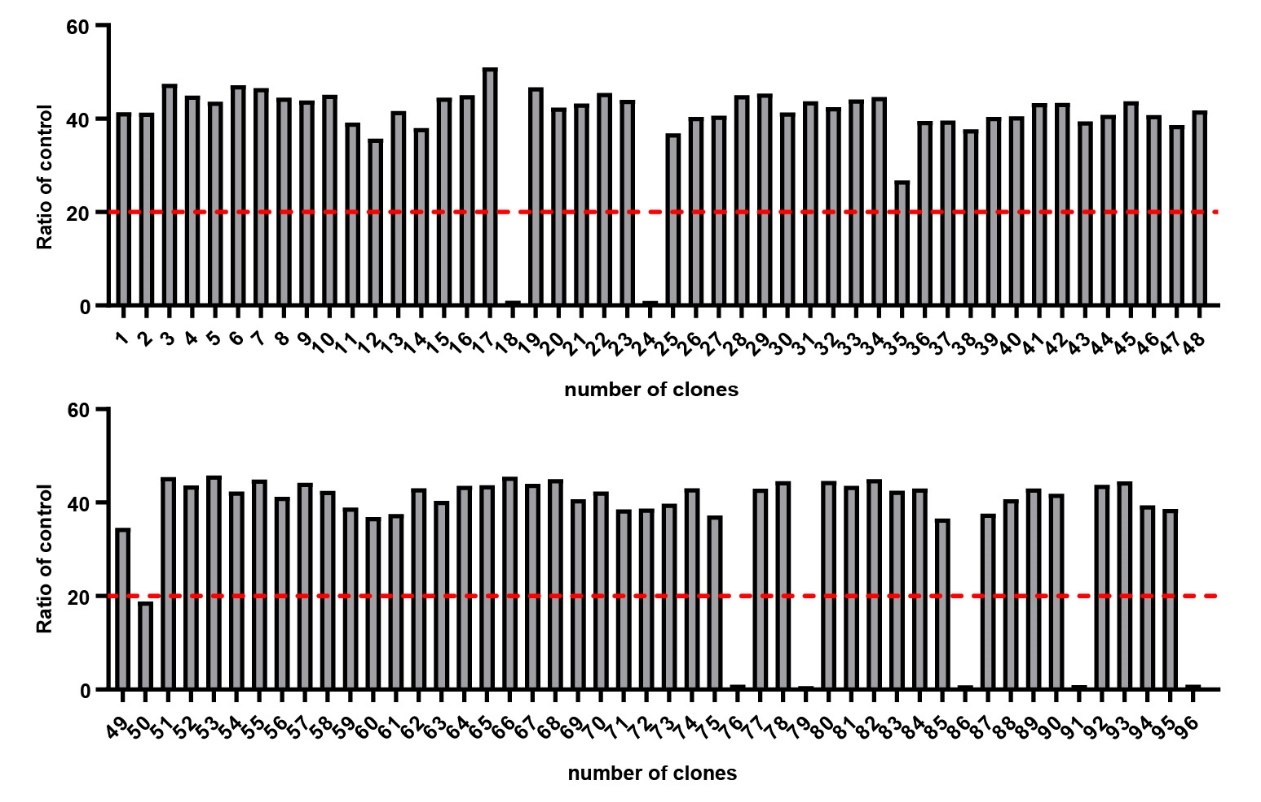


**Figure S2** 88 positive clones were identified through PE-ELISA out of 96 randomly selected clones.


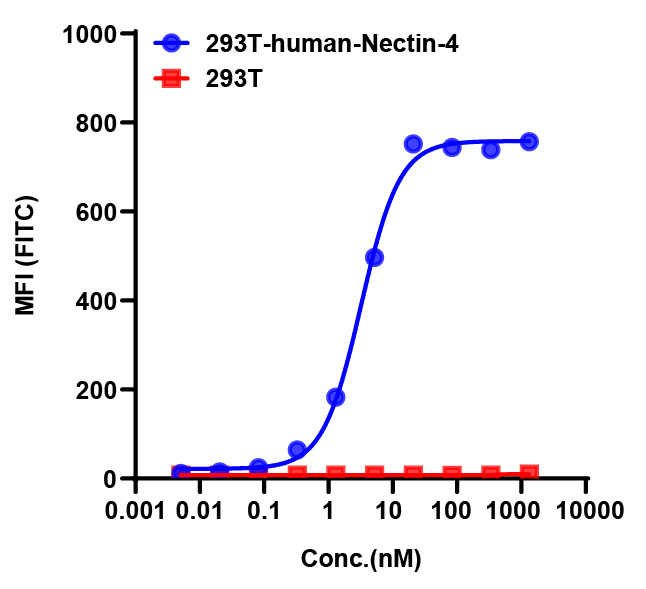


**Figure S3** 293T-human-Nectin-4, which stably expresses the Nectin-4 receptor, was constructed while its over-expression function was confirmed by flow cytometry.
